# Retraction Note: Liver X receptors constrain tumor development and metastasis dissemination in PTEN-deficient prostate cancer

**DOI:** 10.1038/s41467-018-04653-3

**Published:** 2018-07-04

**Authors:** Anthony Alioui, Julie Dufour, Valerio Leoni, Anke Loregger, Martina Moeton, Luigi Iuliano, Chiara Zerbinati, Amandine Septier, Pierre Val, Allan Fouache, Vincenzo Russo, David H. Volle, Jean-Marc A. Lobaccaro, Noam Zelcer, Silvère Baron

**Affiliations:** 1 Université Clermont Auvergne, Génétique Reproduction et Développement, BP 38, 63001 Clermont-Ferrand, France; 2 CNRS, UMR 6293, Génétique Reproduction et Développement, F-63001 Clermont-Ferrand, France; 3 INSERM, UMR 1103, Génétique Reproduction et Développement, F-63001 Clermont-Ferrand, France; 4 Centre de Recherche en Nutrition Humaine d’Auvergne, F-63001 Clermont-Ferrand, France; 5 Department of Medical Biochemistry, Academic Medical Center, Amsterdam, 1105 AZ The Netherlands; 6 Laboratory of Clinical Chemistry, Hospital of Varese, AST_Settelaghi, Varese, Italy; 7 Department of Medico-Surgical Sciences and Biotechnology, Sapienza University of Rome, Latina, 04100 Italy; 8 Unit of Immuno-Biotherapy of Melanoma and Solid Tumors, Division of Experimental Oncology, San Raffaele Scientific Institute, Milan, Italy

**Keywords:** Cancer metabolism, Prostate

Retraction of: *Nature Communications* 10.1038/s41467-017-00508-5, published online 05 September 2017

In this Article, we reported that liver X receptors constrain metastatic development of prostate cancer in Pten-null mice.

However, following institutional investigations by Université Clermont Auvergne, it has come to our attention that much of the data reported in the paper were a result of manipulation or fabrication. Specifically, differences in protein expression in western blots presented in Fig. 1e, 3b, g, i, m, and 6b, and Supplementary Figures 2d, 6d, 6h, 7h, 8c, 8d, and 14 were generated by unequal loading of samples. In qPCR experiments presented in Figs. 1f, 3f, 4g, 5h, and 6a, d and Supplementary Figures 2c, 5d, 6g, 8d, 11a-c, and 12b, differences in expression level were manipulated through adjustment of cycle numbers, selection of samples, and data fabrication. Differences in immunostaining in Figs. 2g, k and 7e were obtained by selection of images or manipulation of exposure levels. Observed differences in relative luminescence units in Figs. 3m, 4e, and Supplementary Figures 6f, 7a, 7b, 10, and 11f were due to experiment selection.

In light of these findings, we have no confidence in the accuracy of the reported data and the conclusions of the paper. We therefore wish to retract the paper. We deeply regret these circumstances and apologize to the scientific community.

